# Redox regulation of the immune response

**DOI:** 10.1038/s41423-022-00902-0

**Published:** 2022-09-02

**Authors:** Gerwyn Morris, Maria Gevezova, Victoria Sarafian, Michael Maes

**Affiliations:** 1 Deakin University, IMPACT – the Institute for Mental and Physical Health and Clinical Translation, School of Medicine, Barwon Health, Geelong, VIC Australia; 2 Department of Medical Biology, Medical University–Plovdiv, Plovdiv, Bulgaria; 3 Research Institute at Medical University–Plovdiv, Plovdiv, Bulgaria; 4 Department of Psychiatry, Faculty of Medicine, King Chulalongkorn Memorial Hospital, Bangkok, Thailand; 5 Department of Psychiatry, Medical University of Plovdiv, Plovdiv, Bulgaria

**Keywords:** Oxidative and nitrosative stress, Immune response, Inflammation, Antioxidants, Physiological stress, Chronic inflammation, Cell signalling

## Abstract

The immune-inflammatory response is associated with increased nitro-oxidative stress. The aim of this mechanistic review is to examine: (a) the role of redox-sensitive transcription factors and enzymes, ROS/RNS production, and the activity of cellular antioxidants in the activation and performance of macrophages, dendritic cells, neutrophils, T-cells, B-cells, and natural killer cells; (b) the involvement of high-density lipoprotein (HDL), apolipoprotein A1 (ApoA1), paraoxonase-1 (PON1), and oxidized phospholipids in regulating the immune response; and (c) the detrimental effects of hypernitrosylation and chronic nitro-oxidative stress on the immune response. The redox changes during immune-inflammatory responses are orchestrated by the actions of nuclear factor-κB, HIF1α, the mechanistic target of rapamycin, the phosphatidylinositol 3-kinase/protein kinase B signaling pathway, mitogen-activated protein kinases, 5' AMP-activated protein kinase, and peroxisome proliferator-activated receptor. The performance and survival of individual immune cells is under redox control and depends on intracellular and extracellular levels of ROS/RNS. They are heavily influenced by cellular antioxidants including the glutathione and thioredoxin systems, nuclear factor erythroid 2-related factor 2, and the HDL/ApoA1/PON1 complex. Chronic nitro-oxidative stress and hypernitrosylation inhibit the activity of those antioxidant systems, the tricarboxylic acid cycle, mitochondrial functions, and the metabolism of immune cells. In conclusion, redox-associated mechanisms modulate metabolic reprogramming of immune cells, macrophage and T helper cell polarization, phagocytosis, production of pro- versus anti-inflammatory cytokines, immune training and tolerance, chemotaxis, pathogen sensing, antiviral and antibacterial effects, Toll-like receptor activity, and endotoxin tolerance.

## Introduction

The instigation of the innate immune response commences as a result of the recognition of an invading pathogen by organ-specific resident macrophages, dendritic cells (DCs), fibroblasts, pericytes, and in many cases endothelial cells [1–4]. This recognition is accomplished by cytosolic or membrane-bound Toll-like or NOD-like pattern-recognition receptors (PRR) that leads to the activation of these sentinel cells and the release of cytokines and chemokines [3–5]. Once secreted these molecules activate endothelial cells that then express chemokines and adhesion factors [6, 7]. Recruitment, binding, and activation of neutrophils, monocytes, macrophages, and platelets follow these processes in turn allowing the migration of myeloid cells into tissues that reach the sites of infection [8–10].

The multiple phenotypical and functional roles of myeloid cells are enabled by metabolic reprogramming comprising of changes in levels of glycolysis, fatty acid oxidation (FAO), the tricarboxylic acid (TCA) cycle activity, involvement of the pentose phosphate pathway (PPP), and mitochondrial respiration [11–13]. This is also true for neutrophils, T-cell activation and differentiation into helper, effector, and cytotoxic subsets [14], B-cell activation, differentiation and antibody production [15], and the activation and cytotoxic properties of natural killer (NK) cells [16].

These metabolic and redox changes are orchestrated and regulated by the cooperative and/or antagonistic actions of nuclear factor (NF-κB), HIF1α, the mechanistic target of rapamycin (mTOR), and the phosphatidylinositol 3-kinase (PI3K)/protein kinase B (AKT) signaling pathway. Mitogen-activated protein (MAP) kinases, 5' AMP-activated protein kinase (AMPK), and peroxisome proliferator-activated receptor (PPAR) are also implicated. All these factors lead to the increase in reactive oxygen species (ROS) produced by mitochondria and to the upregulation of nicotinamide adenine dinucleotide phosphate (NADPH) oxidase (NOX). These transcription factors and enzymes are all redox-sensitive as is the performance of mitochondria [17–23].

In addition, the functioning of individual immune cells is under redox control. It is sensitive to intracellular and extracellular levels of nitric oxide (NO) [24, 25] and ROS [26–28] and is also heavily influenced by the activity of nuclear factor erythroid 2-related factor 2 (Nrf-2) and cellular antioxidants [29–31]. The action of individual immune cells is regulated by oxidized phospholipids [32–35], high-density lipoprotein (HDL), apolipoprotein A1 (ApoA1), paraoxonase-1 (PON1) activity [36–38], and indoleamine 2, 3-dioxygenase (IDO) [39, 40]. The levels and immune functions of these molecular players are under redox control as well [41].

**Fig. 1:**
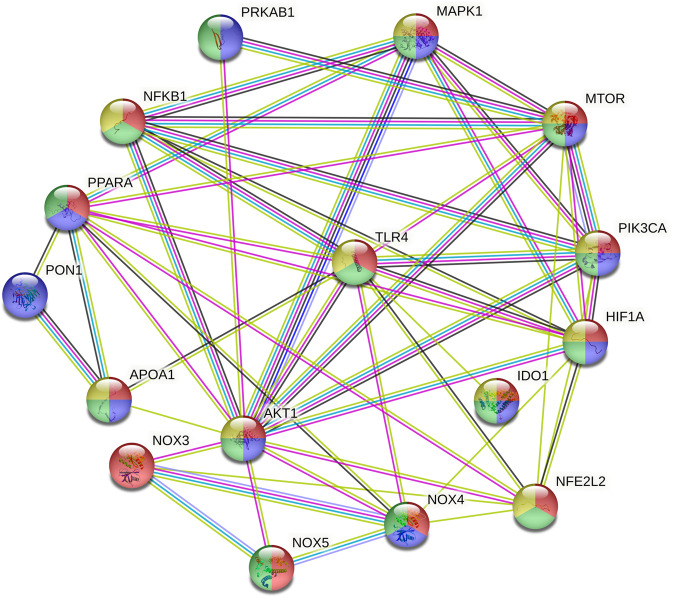
STRING protein–protein network analysis performed on the key proteins included in the present review. Nodes indicate proteins and edges indicate protein–protein interactions. Red colour of the nodes: reflects response to stress (*p* < 1.57E–05), blue node colour: small molecular metabolic process (*p* < 1.68E–05), green node colour: positive regulation of metabolic process (*p* < 2.17E–05), and yellow node colour: regulation of immune system process (*p* < 3.78E–05). Colours of the edges: see https://string-db.org for details. The figure displays the gene names and Table 1 specifies the names and functions of the proteins. NFKB1 nuclear factor (NF)-κB (NF-κB), HIF1A hypoxia-inducible factor 1-alpha (HIF1α), MTOR the mechanistic target of rapamycin (mTOR), PIK3CA phosphatidylinositol 3-kinase (PI3K), AKT1 protein kinase B, MAPK mitogen-activated protein kinases, PRKAB1 AMP-activated protein kinase (AMPK), PPARA peroxisome proliferator-activated receptor, NOX NADPH oxidase, NFE2L2 nuclear factor erythroid 2-related factor 2 (Nrf-2), APOA1 apolipoprotein A1 (ApoA1), PON1 paraoxonase-1, IDO1 indoleamine 2, 3-dioxygenase (IDO), TLR-4 Toll-like receptor-4

Figure 1 shows the outcome of a STRING (STRING version 11.0; https://string-db.org) protein–protein network analysis performed on the aforementioned proteins and enzymes, which are discussed in detail in this review. The zero-order network consists of 16 nodes. The number of edges (*n* = 50) exceeds the expected number of edges (*n* = 13) with *p*-enrichment value of 2.22E–15, average node degree = 6.25 and average local clustering coefficient = 0.78.

Table 1 summarizes the functions of the proteins in this highly interconnected protein interaction network.

**Table 1:** Names and functions of the key proteins included in the present review

ID	Names	Main functions (based on UniProt)	References
NF-κB	Nuclear factor NF-kappa-B	Pleiotropic transcription factor and endpoint of a series of signal transduction events including immune activation, differentiation, cell growth, and apoptosis	[42–44, 88, 89]
HIF1α	Hypoxia-inducible factor 1-alpha	Transcriptional regulator of the response to hypoxia. Activates over 40 genes, e.g., glycolytic enzymes, glucose transporters, vascular endothelial growth factor, and protein that increases oxygen delivery	[47, 49, 54–57]
mTOR	Mechanistic target of rapamycin	In response to stress, hormonal and energy signals, regulates cellular metabolism, survival, and growth	[72–78, 180, 228]
PI3K	PI3-kinase Phosphatidylinositol 4,5-bisphosphate 3-kinase catalytic subunit alpha isoform Phosphatidylinositol 3-kinase	Group of signal transducer enzymes which regulate cellular functions including proliferation, differentiation, survival, motility, and morphology	[45, 47–49, 130, 230, 231]
AKT1	RAC-alpha serine/threonine-protein kinase	Regulates metabolism, cell survival, proliferation, and growth	[22, 46, 48, 73, 331, 479]
MAPK1	Mitogen-activated protein kinase	Mediates adhesion, cell growth, survival, and differentiation via transcription and translational processes and cytoskeletal rearrangements	[435]
AMPK	5'-AMP-activated protein kinase	In response to lowered ATP, regulates energy metabolism and attenuates energy-consuming processes. AMPK reduces carbohydrate, lipid, and protein synthesis	[21, 133, 134, 293, 346, 448]
PPAR	Peroxisome proliferator-activated receptor	Regulates the beta-oxidation pathway and lipid metabolism	[41, 90, 138, 139]
NADPH oxidase (NOX)	Nicotinamide adenine dinucleotide phosphate oxidase	May constitutively produce superoxide	[144, 216, 470, 472]
TLR-4	Toll-like receptor-4	Mediates the immune response to lipopolysaccharides	[85, 87]
Nrf-2	Nuclear factor erythroid 2-related factor 2	Transcription activator that binds to antioxidant response elements in the promoter regions of (antioxidant) target genes	[160, 195, 196, 436]
PON1	Paraoxonase/arylesterase 1	Protects low-density lipoproteins against oxidative modification and consequent atherogenicity	[525, 526]
IDO	Indoleamine 2,3-dioxygenase 1	Catalyzes the first step of the catabolism of tryptophan into kynurenine and other tryptophan catabolites	[535]
ApoA1	Apolipoprotein A-I	Acts as a cofactor for lecithin cholesterol acyltransferase and participates in the reverse cholesterol transport	[522–524]

This paper has three aims. Firstly, to detail the role of redox-sensitive transcription factors and enzymes, ROS, and reactive nitrogen species (RNS) production and the effect of cellular antioxidants on the activation and performance of macrophages, DCs, neutrophils, T-cells, B-cells, and NK-cells. Secondly, to explain the involvement of HDL, ApoA1, PON1, and oxidized phospholipids in regulating the immune-inflammatory response. Thirdly, to clarify the detrimental effects of chronic oxidative and nitrosative stress on the performance of individual immune cells and the immune-inflammatory response as a whole. We will begin with a discussion of the effects of these factors on macrophage activation and function, which offers a vehicle to illustrate many of the principles involved in metabolic reprogramming and the effects of individual signaling molecules, thus avoiding unnecessary repetition in later sections of the paper.

## Metabolic reprogramming and redox factors involved in macrophage activation

### Metabolic reprogramming in macrophages

Macrophages may be activated by cytokines, ROS, and PRR engagement by pathogen-associated molecular patterns, damage-associated molecular patterns, and commensal LPS leading to the activation of NF-κB [42–44] and the PI3K/AKT signaling pathway [45, 46]. Upregulated NF-κB results in increased transcription of proinflammatory cytokines and chemokines, inducible NO synthase (iNOS), and HIF1α [42–44]. Enhanced PI3K signaling also leads to the upregulation of mTOR [47–49] which in turn reinforces the upregulation of HIF1α [45, 46]. These signaling pathways, enzymes, and transcription factors play an essential role in maintaining macrophage activation and M1 polarization by driving metabolic reprogramming. It involves the downregulation of ATP production by mitochondrial oxidative phosphorylation (OXPHOS) and FAO [50, 51] to ATP production via aerobic glycolysis [52].

The shift to aerobic glycolysis is an indispensable metabolic event for M1 macrophages in terms of maintaining and increasing phagocytosis, production of ROS and proinflammatory cytokines and unsurprisingly, its inhibition may impair those functions [53–55]. Maintenance of this state is dependent on the activity of a range of transcription factors, most notably mTOR and HIF1α, with the latter playing a dominant role in enabling the continuance of glycolysis under normoxic conditions [49, 56].

HIF1α acts as a modulator of transcription by changing the methylation status of hypoxia-responsive elements in the promoter regions of target genes involved in the termination of OXPHOS and the instigation of aerobic glycolysis [57]. For example, HIF1α upregulation suppresses the activity of electron transport chain (ETC) enzymes [58, 59], decreases mitochondrial activity, and induces mitochondrial autophagy [60, 61]. Increased activity of this transcription factor also suppresses genes involved in FAO [62, 63]. HIF1α restrains metabolism by activating the gene for pyruvate dehydrogenase kinase 1, which in turn inhibits the TCA cycle [64] and inactivates pyruvate dehydrogenase [65]. In addition, HIF1α-regulated gene expression reduces the production of acetyl-CoA and succinyl-CoA [66].

HIF1α intensifies glycolytic flux, thereby augmenting the expression of glucose transporters (GLUT-1 and GLUT-3) [67]. Glycolysis is stimulated by the high levels of hexokinases [68], aldolase A, enolase 1 [69], and phosphoglycerate kinase 1 [70]. Finally, HIF1α also induces the transcription of lactate dehydrogenase A, which plays an indispensable role in maintaining a continuous supply of NAD^+^, thereby enabling the continuation of glycolysis [71]. HIF1α-regulated gene expression prevents acetyl-CoA from being synthesized from glucose and fatty acid-derived carbons [66].

While the role of HIF1α in instigating and regulating the transition between OXPHOS and aerobic glycolysis is of paramount importance, it should be emphasized that the activation of mTOR is involved. Firstly, mTOR stabilizes and enhances the activity of HIF1α and, secondly, it increases the rate of glycolysis, AKT, forkhead box transcription factors (FoxO), hexokinase II, and Myc proto-oncogene [72–74]. Upregulated mTOR participates in further reducing OXPHOS by enhancing NO and interferon (IFN)-γ production, thus compromising the activity of the mitochondrial ETC [75]. In total, the actions of mTOR inhibit M2 polarization [76] and stimulate M1 polarization [77, 78].

The PPP main role is to utilize the energy released from the metabolism of glucose-6-phosphate into ribulose-5-phosphate to form NADPH. The latter is used in the production of NADPH oxidase and as a reducing equivalent enabling the function of the glutathione (GSH) and thioredoxin antioxidant systems [13, 79]. The activation of M1 polarized macrophages also results in several other aspects of metabolic reprogramming in order to maintain the inflammatory status and prolong survival. Most notable are the upregulation of the cytosolic PPP [50, 80], increased lipid synthesis, and decreased lipid catabolism [62, 81], altered glutamine and arginine metabolism [81, 82], and a “broken” TCA cycle [83, 84]. These parameters are discussed below commencing with the Toll-like Receptor (TLR) and proinflammatory cytokine-mediated reprogramming of the lipidome [85].

The synthesis of lipids is a key component in membrane remodeling. In M1 macrophages the process depends on the production of acetyl-CoA from citrate ATP-citrate lyase [86]. The activity of this enzyme rapidly increases in activated macrophages. Intracellular fatty acids can also be used to synthesize triglycerides for energy storage, and sphingolipids for membrane synthesis, as well as eicosanoids for signaling [81]. The increase in lipid synthesis is largely enabled and regulated by the high activity of sterol regulatory element binding protein-1 (SREBP-1) by TLR-4 and PI3K-activated mTOR [73, 87]. It is also controlled by the enhanced expression of NF-κB and the presence of proinflammatory cytokines [88, 89]. SREBP-1 activation stimulates the synthesis of proinflammatory cytokines, ROS, and triggers the inflammasome [87–89]. M1 activation is accompanied by elevated iNOS, which induces the conversion of arginine to NO, so that the production of other RNS may be initiated [82, 90, 91].

M1 polarized macrophages accumulate cytosolic citrate stemming from the decreased activity of isocitrate dehydrogenase (IDH) [50] and the upregulation of the mitochondrial citrate carrier (CIC) [92, 93]. The increased activity of IDH is mediated by ADP levels [94]. CIC is upregulated by several inflammatory mediators such as tumor necrosis factor (TNF)-α, IFN-γ, or commensal LPS via the upregulation of NF-κB and or STAT-1 [92, 95]. In this scenario, citrate exerts a multiplicity of vital roles, enabling macrophage function and inflammatory status such as increasing NO, ROS, and prostaglandin E2 (PGE2) production [92, 96]. Cytosolic citrate can also act as a source of NADPH, either as a result of malate import into mitochondria via CIC, and the subsequent formation of pyruvate via malic enzyme, or the conversion of citrate into alpha-ketoglutarate via the action of cytosolic IDH [97, 98]. Cytosolic citrate is also a substrate of ACLY, producing acetyl-CoA and oxaloacetate and upregulating acetyl-CoA carboxylase (ACC) stimulating lipid synthesis [99].

Activated M1 polarized macrophages are characterized by high levels of cytosolic itaconate from cis-aconitate drawn from the Krebs cycle via a significant inflammation-mediated upregulation of macrophage aconitate decarboxylase 1 [100, 101]. Itaconate is involved in tolerance and suppression of inflammation [102, 103], inhibits mitochondrial respiration, stabilizes HIF1α, and activates Nrf-2 via alkylation of KEAP-1 [84, 104]. Finally, itaconate accumulation leads to the inhibition of succinate dehydrogenase, directing the accumulation of succinate and leading to numerous proinflammatory and prooxidative consequences [103, 105, 106]. For example, elevated succinate oxidation in a cellular environment of few or no ATP generation induces a phenomenon described as reverse electron transport whereby electrons flow “backwards” along the ETC to complex I. As a result, large increases in the genesis and release of ROS follow [107, 108]. High levels of cytosolic succinate may induce an increase in lysine group succinylation in the cellular proteome, which many influence protein activity via changes in charge and conformation [109]. The mechanisms involved are beyond the scope of this review, but it is important to note that this post-translational modification offers another route relaying subtle redox-mediated metabolic changes to protein function [110]. Finally, once externalized, succinate can bind to the G protein-coupled succinate receptor 1 (SUCNR1) that is expressed on the surface of activated M1 polarized macrophages [111, 112]. This is a mechanism involved in sustaining and amplifying their inflammatory effects [12, 113].

### M2 polarized macrophages

In an environment of elevated IL-4 and or IL-13, activated M1 polarized macrophages may ultimately be driven toward a range of anti-inflammatory and tissue healing phenotypes classified as M2a, M2b, M2c, and M2d that for the purposes of this paper may be usefully described as “M2” [114–116]. Tyrosine phosphorylation and activation of the signal transducer/transcription activator 6 (STAT-6) are required for macrophage M2 polarization [117, 118]. The latter then triggers a wide range of M2-associated genes including GATA binding protein 3 (GATA3), CD36, arginase-1 (*Arg1*), matrix metalloproteases (MMPs), FIZZ1, and PPARγ [119, 120]. IL-4 and IL-13 also upregulate the activity of transforming growth factor (TGF)-β, suppressor of cytokine signaling 1 (SOCS-1), and insulin-like growth factor 1 (IGF-1) that act to suppress the production of proinflammatory cytokines and promotes tissue repair [114, 115, 121]. Unlike M1 polarization, M2 polarization is associated with a return to OXPHOS and increased FAO [114, 115]. In addition, M2 polarized macrophages possess an intact TCA cycle [114, 115].

M2 macrophages are also characterized by activation of the nuclear liver X receptor (LXR) thereby regulating lipid synthesis and cholesterol homeostasis [122]. Overexpression of LXR inhibits NF-κB and activator protein-1 (AP-1) to reduce M1 responses and inflammation [123, 124]. One major element reinforcing the transition from M1 to M2 polarization is the change in the metabolism of arginine. In M1 polarized macrophages, elevated activity of iNOS leads to the metabolism of arginine to produce citrulline and NO. The latter is a major element in maintaining the switch toward aerobic glycolysis as explained above [84]. However, in M2 polarized macrophages, the increased transcription of arginase-1 metabolizes arginine to ornithine and urea. They both play a vital role in M2 macrophage survival, proliferation, and tissue repair [120, 125]. Glutamine metabolism is also of particular importance in M2 macrophages for two main reasons. Firstly, oxidation of this amino acid is an essential source of acetyl-CoA in an inflammatory environment leading to depleted extracellular glucose levels thereby maintaining TCA activity [126–128]. Secondly, glutaminolysis-mediated increase in α-ketoglutarate and the activation of the glutamine–UDP-*N*-acetylglucosamine (GlcNAc) pathway reinforce M2 polarization [126].

There are major differences in the regulation of the metabolic bioenergetic pathways involved in the transition to M2 polarization compared to those governing M1 polarization. In the case of M2 polarization the main players are AMPK and PPARγ whose activities are briefly described below. AMPK stimulates OXPHOS and FAO while inhibiting NF-κB and mTOR. This, in turn, decreases inflammation, reduces the levels of HIF1α, and terminates aerobic glycolysis [129–132]. AMPK inhibits ACC, increases glycolytic flux, mitogenesis, lipases, autophagy, and lysosomal degradation [133, 134]. PPAR-γ upregulates FAO, maintains mitochondrial membrane potential, mitochondrial citrate synthase, and regulates numerous genes involved in mitochondrial function including transcription factor A (TFAM), and peroxisome proliferator-activated receptor-gamma (PGC)-1α [135–138]. It also downregulates NF-κB and upregulates Nrf-2 [135–137]. PPAR stimulates the activity of LXR [139], which controls cholesterol and lipid homeostasis. Thus, inflammation is reduced and glycolysis is blocked via the inhibition of NF-κB [123, 124]. Finally, PPAR-γ promotes the oxidation of glutamine [126] whose importance in M2 polarization has been discussed above [140].

### Redox regulation of macrophage activation functions and survival

Macrophage ROS levels affect the activity of STAT-1, MAPKs, and NF-κB and lead to an overall increase in inflammatory signaling [141]. ROS levels also affect the assembly of NADPH oxidase subunits and regulate the formation of corrosive RNS species such as peroxynitrite, thereby influencing H_2_O_2-_mediated intracellular signaling and macromolecule damage [142]. Continually high ROS or NO levels are accompanied by the development of macrophage senescence [143–145]. The mechanisms driving this phenomenon appear to involve the persistent expression of NF-κB, STAT-3, IL-10, and TGF-β, and potentially the upregulation of PD-1 [144, 146, 147].

There is also ample evidence that macrophage functions and polarization patterns are influenced by GSH levels and the overall activity of the GSH system [148, 149]. For example, increased GSH oxidation compromises phagocytosis and macrophage survival [150, 151]. The GSH system also plays a key role in regulating M1 inflammatory status and the production of PGE2 and NO, while protecting macromolecules from oxidative damage [152, 153]. The antiviral responses initiated following M1 macrophage activation such as increased expression of STAT-1, Irf7, and Irf9 are also dependent on an optimally functioning GSH system and are compromised by GSH depletion [154].

Thioredoxin (TRX)-1 affects the inflammatory status of macrophages by modulating the activity of macrophage receptors, and the macrophage migration inhibiting factor (MIF) [155]. The latter effect reduces the proinflammatory status of M1 macrophages and encourages M2 polarization by lowering TNF-α and monocyte-chemoattractant protein (MCP)-1 production [156–159].

Nrf-2 upregulation also exerts an anti-inflammatory effect in activated macrophages by attenuating the activity of IL-1β and IL-6 [160, 161]. The mechanism involves Nrf-2 binding at the relevant gene promoter sites resulting in inhibition of the recruitment of RNA Polymerase II complex [162]. Nrf-2 upregulation also rises the expression of CD163 and Arg1 [161, 163]. It affects the transcription of a multitude of genes involved in the switch between M1 and M2 polarization [160, 161].

**Fig. 2:**
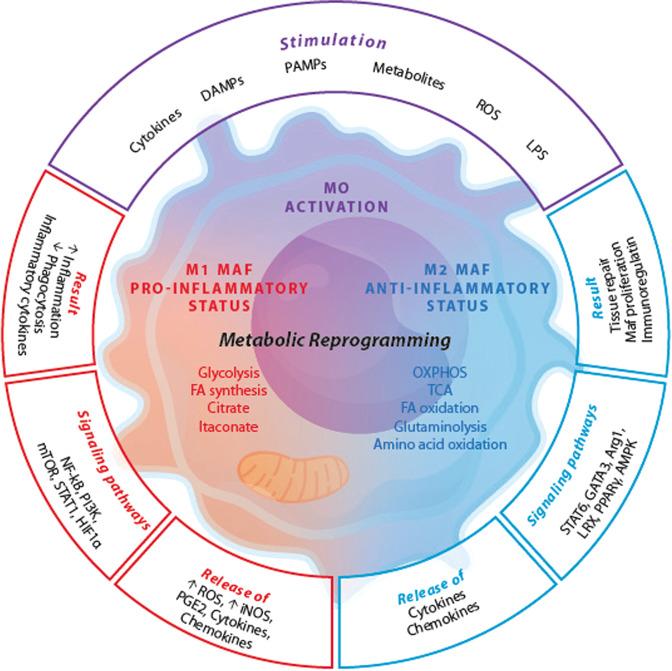
Metabolic reprogramming in macrophages (Maf). DAMPs damage-associated molecular patterns, PAMPs pathogen-associated molecular patterns, ROS reactive oxygen species, LPS lipopolysaccharide, STAT-6 signal transducer/transcription activator 6, GATA3 GATA binding protein 3, Arg1 Arginase-1, LXR liver X receptor, PPARγ peroxisome proliferator-activated receptor, AMPK AMP-activated protein kinase, iNOS inducible nitric oxide synthase, NO nitric oxide, PGE2 prostaglandin E2, OXPHOS oxidative phosphorylation, TCA tricarboxylic acid cycle, FA fatty acid, NF-kB nuclear factor NF-kappa-B, PI3K phosphatidylinositol 3-kinase, mTOR mechanistic target of rapamycin, STAT-1 signal transducer and activator оf transcription 1, HIF1α hypoxia-inducible factor 1-alpha

The metabolic reprogramming in macrophages is presented in Fig. 2 and Table 2 summarizes the effects of redox mechanisms on macrophage functions.

**Table 2:** Redox mechanisms influencing macrophage functions

Redox mechanisms	Macrophage functions	References
Reactive oxygen species (ROS)	Increase inflammatory signaling via STAT-1, MAPK, and NF-KB mechanisms	[141]
	Modulate NADPH oxidase assembly thereby further increasing superoxide and ROS production as well as RNS with peroxynitrite formation	[142–147]
Glutathione (GSH)	GSH oxidation compromises phagocytosis and leads to attenuated macrophage survival	[148–151]
	Regulates M1 inflammatory status	[152, 153]
	As a ROS scavenger, protects against oxidative stress damage	[154]
Thioredoxin (TRX)	Modulates MIF signaling thereby lowering inflammation and encouraging M2 polarization	[155–159]
Nuclear factor erythroid 2-related factor 2 (Nrf-2)	Anti-inflammatory effects through attenuating IL-1 and IL-6	[160–163]
	Transcription of a multitude of genes involved in the switch between M1 and M2 polarization	[160, 161]

## Metabolic reprogramming and redox factors involved in Dendritic cells activation

### Metabolic reprogramming of DCs

DCs are archetypal antigen presenting cells (APCs) and play a dominant role in linking innate and humoral immunity [164]. In physiological conditions, tissue-resident DCs drain to the lymph nodes and, thereafter, present self-antigens to T-cells, thereby maintaining immune tolerance [165]. However, after pathogen invasion, TLR- mediated activation of DCs is followed by numerous changes in function and phenotype resulting in their active migration to lymph nodes and cytokine production [166].

Resting-state DCs rely on OXPHOS-driven TCA cycle activity fueled by glutaminolysis and FAO to meet their energy needs [167, 168]. Their overall metabolism is regulated by AMPK [168]. However, following pathogen recognition, TLR engagement results in activation of NF-κB, PI3K/AKT signaling, mTOR, and PPAR-γ and in a rapid shift to aerobic glycolysis and lactate production in a similar manner to M1 polarized macrophages discussed above [169, 170]. In addition, glycolytic intermediates are shunted into the PPP while increased NO production inhibits the ETC. Moreover, citrate is withdrawn from the TCA acting as a crucial player in FA synthesis that maintains and increases inflammatory cytokines, NO, and ROS production [171, 172]. The acute switch to glycolytic metabolism is facilitated by PI3K /AKT signaling [173]. However, chronic aerobic glycolysis is enabled and regulated by mTOR and HIF1α activation [174, 175]. In addition, upregulation of mTOR and the subsequent increase in HIF1α activity induces the transcription of iNOS [176, 177] leading to NO-mediated suppression of mitochondrial OXPHOS via reversible inhibition of ETC complex I, III, and IV [17, 178, 179]. mTOR activation initiates and controls lipid synthesis and mitochondrial biogenesis via the downstream upregulation of SREBPs and PPAR. It stimulates IL-6, IL-1, and TNF-α production, via the upregulation of AKT, FOXO3, and Myc [180]. mTOR activation serves as the enabler and master regulator of DC migration, maturation, and endocytosis [180].

### Redox regulation of DC activation and function

Phagosomal ROS levels are involved in MH1-mediated presentation of digested antigens to CD8 T cells [181, 182]. In this context, it is noteworthy that the activation of CD8 T cells requires upregulation of mitochondrial reactive oxygen species (mtROS) production [183]. DC production of ROS following TLR activation also plays a major role in the maturation and priming of CD4 T cells [184, 185]. Many aspects of DC function are influenced by the GSH system activity. For example, GSH levels regulate DC differentiation and function as APCs [186]. DC GSH levels also determine T-cell polarization patterns by affecting IL-27 and IL-12 production [187, 188]. GSH depletion is associated with the differentiation of naive T cells [188] and inhibits DC maturation and inflammatory cytokine production leading to profound cellular dysfunction [189]. Moreover, DCs directly influence the redox state of activated T cells via the transfer of thioredoxin [190].

Redox homeostasis within activated DCs is regulated by Nrf-2 which acts to restrain T-cell proliferation by repressing IL-12 production and upregulating IL-10 [191, 192]. Conversely, DCs that lack Nrf-2 generate increased numbers of activated T helper (Th) cells and reduced numbers of T regulatory (Treg) cells [193]. Moreover, Nrf-2 depletion and the resultant prooxidative state in DCs encourage a Th-2 pattern of differentiation in naive T cells [194, 195]. Finally, Nrf-2 also plays an important role in the transition between glycolysis and OXPHOS in tolerogenic DCs that enables their long-term survival [196].

There is considerable evidence of DC dysfunction in diseases underpinned by chronic inflammation and oxidative stress [197, 198]. Such dysfunction may be directly or indirectly driven by increased inflammatory cytokines, RNS, and ROS. Direct effects involve damage to functional macromolecules and increased activation of apoptotic pathways [199, 200]. Indirect effects include enhanced Wnt signaling [90], epigenetic dysregulation, and compromised TLR activity [166, 201–203].

**Fig. 3:**
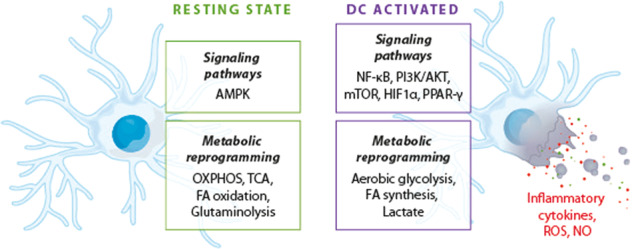
Metabolic reprogramming of dendritic cells (DCs). OXPHOS oxidative phosphorylation, TCA tricarboxylic acid cycle, FA fatty acid, NF-kB nuclear factor NF-kappa-B, mTOR mechanistic target of rapamycin, HIF1α hypoxia-inducible factor 1-alpha, PPARγ peroxisome proliferator-activated receptor, ROS reactive oxygen species, NO nitric oxide

The metabolic reprogramming of DCs is shown in Fig. 3 and Table 3 summarizes the effects of redox mechanisms on DC functions.

**Table 3:** Redox mechanisms influencing dendritic cell and neutrophil functions

Redox mechanisms	Dendritic cell functions	References
Reactive oxygen species (ROS)	ROS due to NADPH oxidase (NOX-2) modulates the presentation of digested antigens to CD8 T cells	[181–183]
	ROS due to TLR activation modulates maturation and the priming of CD4 T cells	[184, 185]
Glutathione (GSH)	DC differentiation and function as APC	[186–189]
	T-cell polarization	[188]
	DC maturation and inflammatory cytokine production	[189]
Nuclear factor erythroid 2-related factor 2 (Nrf-2)	Redox homeostasis in DCs	[190–192]
	Restrains T-cell proliferation by repressing IL-12 production and upregulating IL-10	[191–195]
	Transition between glycolysis and OXPHOS in tolerogenic DCs	[196]
Redox mechanisms	Neutrophil functions	
Reactive oxygen species (ROS)	Compromise initiation and outcome of phagocytosis	[235]
	Dysregulate or decrease oxidative burst and NET production	[236, 237]
	Neutrophil sensing of pathogens	[238, 239]
	Activation of the NLRP3 inflammasome	[238, 239]
Nitric oxide (NO)	Inhibits neutrophil migration, crawling, and adhesion	[240–243]
	Downregulates adhesion molecules	[244]
	Compromises neutrophil binding to the endothelium	[244]
Peroxynitrite	Compromises neutrophil migration	[245–248]
Nuclear factor erythroid 2-related factor 2 (Nrf-2)	Efficiency of neutrophil phagocytosis	[250]
	Recruitment to inflammatory sites and survival	[251, 252]
GSH reductase	Sustains neutrophil respiratory burst	[245, 253]
	Sustains NET production	[253, 254]
	Influences optimal phagocytotic activity	[255, 256]
Thioredoxin (TRX)	Neutrophil chemotaxis	[263, 264]
	Desensitization of neutrophils toward monocyte-chemoattractant protein-1	[264–266]

## Metabolic reprogramming and redox regulation of neutrophil activation

### Metabolic reprogramming of neutrophils

Neutrophils are the first line responders of the innate immune system, which play a key role in the destruction of invading pathogens. However, these leucocytes also participate in humoral immunity via a sophisticated cross-talk with other immune cells [204–206]. Importantly, these regulatory functions extend beyond modulation of the activity of myeloid cells and also involve modifying the function of T-cells, marginal zone B-cells, and NK-cell homeostasis [204–206]. There is also considerable evidence of functionally distinct subsets and extensive cellular plasticity enabling a range of roles depending on cellular location and inflammatory status [207, 208]. These immune cells may be activated and/or primed by multiple stimuli such as inflammatory cytokines, chemokines, growth factors, PRRs (mainly c-type lectin receptors), opsonins (C3a and IgG), and G protein-coupled receptors [209, 210].

Glycolysis is the primary energy source for activated neutrophils under physiological conditions [211]. This is also true for inflammatory environments [212]. However, neutrophils adjust their metabolism to carry out their various effector functions such as phagocytosis, degranulation, oxidative burst, neutrophil extracellular traps (NET) formation, and chemotaxis [213]. The weight of evidence suggests that NET formation is reliant on glycolysis, with extensive involvement of lactate synthesis, the PPP, and glutamine metabolism as sources of NADPH [214, 215]. This metabolic reprogramming also supplies superoxide production, and induces ROS and hypochlorous acid, used in the neutrophil oxidative burst following phagocytosis of invading pathogens [211, 216–218]. The metabolic changes underpinning chemotaxis are somewhat more complicated, however, and involve mitochondrial contributions in addition to upregulated glycolysis [219–221]. This activity supplies ATP which activates membrane-bound P2Y2 receptors following the receipt of chemotactic stimuli (2019–2021). Mitochondrial activity provides the ATP required for neutrophil activity in regions of profound glucose deprivation. It occurs in an environment of extreme inflammation and also plays a dominant role in neutrophil autophagy and survival via FAO (2011) [222].

These metabolic changes underpinning neutrophil activity in inflammatory environments are primarily regulated by the cooperative action of NF-κB [43, 223], HIF1α [224, 225], and mTOR [211, 226]. The multiple and arguably pivotal roles of the latter include the regulation of NET production, autophagy, oxidative burst, phosphorylation, and stabilization of NOX and HIF1α [226, 227]. mTOR also increases the surface expression of GLUT-1 and intensifies mitochondrial biogenesis and FAO via the upregulation of PPARγ and SREBPs [72]. Elevated mTOR activity increases the production of leukotrienes, prostaglandins, resolving, and proinflammatory cytokines via phosphorylation of AKT [228]. mTORC1 also exerts an inhibitory effect on OXPHOS by upregulation of IFN-γ and NO which inhibits the activity of enzymes in the ETC [229].

While mTOR upregulation plays a key role in the optimal function of activated neutrophils, it should be stressed that other enzymes and transcription factors are also important regulatory elements enabling pathogen destruction. This in turn restrains extreme inflammation and prevents excessive survival. For example, PI3K enables chemotaxis and endothelial crawling via an intricate pattern of “cross-talk” with the Rho family GTPases [230, 231]. On the other hand, AMPK regulates and restrains NF-κB and the production of proinflammatory cytokines, limiting tissue inflammation and destruction while optimizing chemotaxis and phagocytosis [232, 233]. Finally, PPAR-γ also regulates migration and restrains inflammation by inhibiting NF-κB while stimulating IL-10 production [211, 234].

### Redox regulation of neutrophil activation and function

The function of individual neutrophils is heavily influenced by cellular redox status in terms of cellular antioxidant system activity and or ROS/RNS production. For example, excessive ROS fabrication may compromise the initiation and outcome of phagocytosis [235], resulting in a dysregulated or decreased oxidative burst [236] and production of NETs [237]. In addition, intracellular and extracellular levels of ROS play a role in neutrophil “sensing “ of pathogens and consequent activation of the NLRP3 inflammasome and cytokine synthesis [238, 239]. Chronically upregulated ROS and cytokine production may also result in the internalization of membrane chemokine receptors, most notably CXCR2 [240], thereby decreasing neutrophil migration.

Upregulated NO inhibits neutrophil migration, crawling, and adhesion [241–243]. Mechanistically, this is achieved via the downregulation of adhesion factors such as E-selectin, P-selectin, ICAM-1, and VCAM-1. As a result, neutrophil binding to the endothelium is compromised, and subsequent crawling and transmigration to inflammatory centers are damaged [244]. Neutrophil migration may also be hampered by increased production of peroxynitrite due to the combination of NO and superoxide cations [245–248]. There is evidence suggesting that the tyrosine nitration mediates inhibition of P-selectins [245–247] and upregulation of haem oxygenase (HO-1)-1 [249].

A multitude of neutrophil functions is heavily affected by the cellular antioxidant system. For example, Nrf-2 activity influences the efficiency of neutrophil phagocytosis [250], recruitment to inflammatory sites [251], and prolonged survival [252]. The glutathione system regulates various functions displayed by activated neutrophils most notably the stimulation of glutathione reductase. It sustains the neutrophil respiratory burst and NET production [253, 254] influencing optimal phagocytic activity [255, 256]. It is noteworthy that the basal activity of the GSH system in neutrophils appears to be lower than that found in myeloid cells [257], rendering these immune cells vulnerable to depleted GSH levels [257]. This may result in compromised cytoskeletal reorganization, affecting chemotaxis and transmigration and leading to reduced recruitment to sites of inflammation, impaired degranulation, and early apoptosis [258, 259]. In this context, it should be noted that prolonged neutrophil activity depletes levels of GSH, likely due to excessive production of myeloperoxidase (MPO) during chronic nitro-oxidative stress and inflammation [260–262].

TRX plays an important role in the regulation of neutrophil chemotaxis as a result of its release from infected cells and/or inflamed tissues [263, 264]. This effect appears to be a result of the desensitization of neutrophils toward MCP-1 [264, 265], thereby restraining neutrophil recruitment into inflammatory tissues [266]. The mechanisms involved are not fully understood, but they appear to rely at least in part on the oxidation state of functional cysteine residues within the TRX protein [264].

**Fig. 4:**
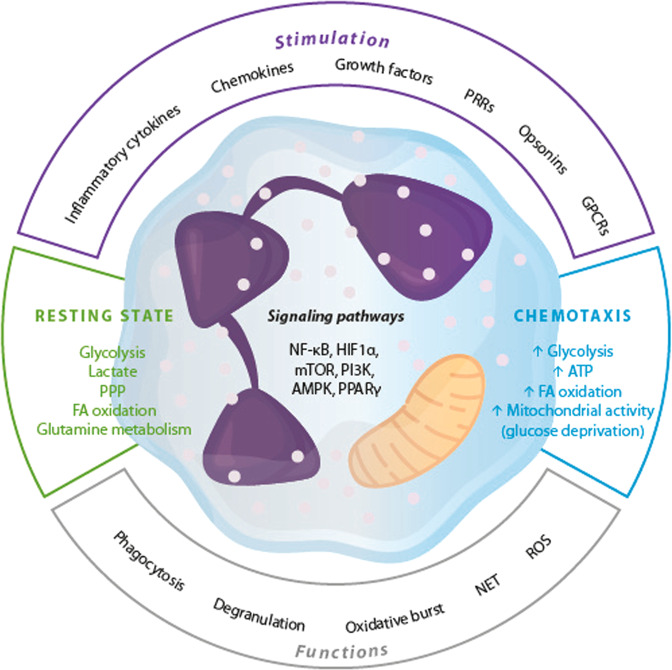
Мodulation of effector functions of neutrophils. PRRs pattern-recognition receptors, GPCRs G protein-coupled receptors, NET neutrophil extracellular traps, ROS reactive oxygen species, PPP pentose phosphate pathway, FA fatty acid, ATP adenosine triphosphate, NF-kB nuclear factor NF-kappa-B, HIF1α hypoxia-inducible factor 1-alpha, mTOR mechanistic target of rapamycin, PI3K phosphatidylinositol 3-kinase, AMPK AMP-activated protein kinase, PPARγ peroxisome proliferator-activated receptor

Table 3 summarizes the redox mechanisms that affect neutrophil functions, and the metabolic reprogramming of neutrophils is presented in Fig. 4.

## Metabolic reprogramming and redox regulation of T-cell activation

### Metabolic reprogramming of T-cells

Activation of T-cells follows the ligation of the T-cell receptor (TCR) and the major histocompatibility complex molecules by APC. Nuclear factor of activated T cell 1 (NFAT1), activation protein-1 (AP)-1, and NF-κB are triggered as a result of this signaling cascade [267]. When TCRs are ligated, ROS production increases by mitochondria and NOXs [268], which in turn regulates the signaling pathways required to enable and modulate T-cell activation, proliferation, and differentiation [268].

Unsurprisingly, T-cell activation and differentiation require extensive metabolic reprogramming [269–273]. In general, such reprogramming is regulated by the collaborative activity of PI3K/AKT, mTOR, HIF1α, and c-Myc [274–276]. However, it should be stressed that the metabolic reprogramming pathways of various T-cell subsets display important differences [277–279]. The metabolic needs of naive and memory T and Treg cells are relatively modest and are met by reliance on OXPHOS and FAO [274, 277, 279]. However, the differentiation and various effector functions of effector CD4 and CD8 cells require ATP obtained from aerobic glycolysis and NADPH. They are supplied by increased activity of the PPP and glutaminolysis, which is largely mediated by high levels of HIF1α and mTOR [278, 280–284].

Important differences exist between subsets when it comes to FA metabolism and T-cell activation and differentiation. For example, effector T-cell activity relies on FA uptake and FAS while T memory cells utilize stored FA [285, 286]. Uniquely, the relative reliance on FA uptake versus FA synthesis exerts a major influence on the differentiation of naive T cells into Tregs or Th-17 cells [286, 287]. In particular, uptake of environmental FA is a characteristic feature of Treg development, while Th-17 differentiation counts on ACC-mediated FA synthesis [276, 287].

TCR signaling also leads to the upregulation of amino acid transporters, facilitating the uptake of branch chain amino acids such as alanine, cysteine, leucine, glycine, and glutamine [288–290]. These amino acids, in combination with high PPP activity, promote the rapid increase of GSH needed for T-cell survival and function [284]. Augmented glutamine catabolism following T-cell activation, mediated by mitochondria-dependent oxidation, is of particular importance as the resultant increase in α-ketoglutarate production stimulates TCA activity and fuels increased OXPHOS [268, 291]. TCR-dependent uptake of glutamine, valine, and leucine is implicated in inflammatory T-cell responses, the differentiation of Th-1 and Th-17 cells, and the development of effector and memory CD8 cells [292–295].

### Redox regulation of T-cells

ROS levels rise rapidly after TCR engagement and are critical in driving T-cell activation, proliferation, and differentiation [268, 291, 296, 297]. Unsurprisingly, given the information discussed above, ROS influences the differentiation patterns and the disparate effector functions of various T lymphocytes. For example, the Th-2 polarized phenotype is encouraged by excessive microenvironmental ROS [298]. Conversely, Th-1 and Th-17 polarizations occur at low microenvironmental levels of ROS [299]. Excessive ROS resulting from either high production or damaged cellular antioxidant defenses may lead to mitochondrial membrane polarization with fatal consequences for T-cell activation and survival following TCR engagement [300]. Similarly, prolonged or chronic ROS upregulation may result in T-cell hyperresponsiveness, exhaustion, and anergy [301–305]. Several mechanisms appear to underpin this phenomenon including compromised mitochondrial ETC activity and dynamics [302, 306], upregulation of PD-1 [307, 308], dysregulated NF-κB signaling, chronic IKKβ signaling [309–311], and oxidation of functional cysteine groups in proteins [312–314]. Finally, excessive ROS production may lead to dysregulated T-cell homeostasis by differential modulation of T-cell homeostasis as effector T cells are more susceptible to ROS-mediated cell death than Tregs [201, 315, 316].

Nrf-2 transcription is upregulated following TCR engagement on naive T cells and restrains inflammatory T-cell activity. Thus, a Th-2 pattern is activated following TCR stimulation [317, 318]. Animal studies show that the upregulation of Nrf-2 increases the proliferation of Tregs [319] and amplifies their immunosuppressive and cytotoxic functions [320].

As previously discussed, GSH synthesis rapidly escalates following TCR activation and affects T-cell survival and function [284]. Increased de novo GSH synthesis also suppresses Th-17 differentiation while encouraging the production of Tregs. Conversely, GSH depletion or loss of de novo GSH synthesis in a state of chronic nitro-oxidative stress [321] compromises mTOR, NFAT, and N-Myc function. Thus, the metabolic reprogramming is abrogated enabling the maintenance of aerobic glycolysis and leading to the termination of T-cell activation [322–324]. Tregs also appear to exert at least some of their cytotoxic and immunosuppressive functions on effector T cells by decreasing GSH synthesis [325].

The TRX system activity exerts a range of influences on T-cell proliferation and activation via increased TRX-1 production. This restrains their stimulation and encourages the development of Tregs from naive T cells, decreasing their differentiation down the Th-1 and Th-17 pathways [326]. TRX-1 upregulation is important in enabling T effector and Treg cell survival and function during chronic nitro-oxidative stress by protecting membrane protein thiols from oxidation [327, 328]. Increased TRX-1 activity is needed to maintain the production of IL-2 [329] and Th-mediated activation of B cells [330].

**Fig. 5:**
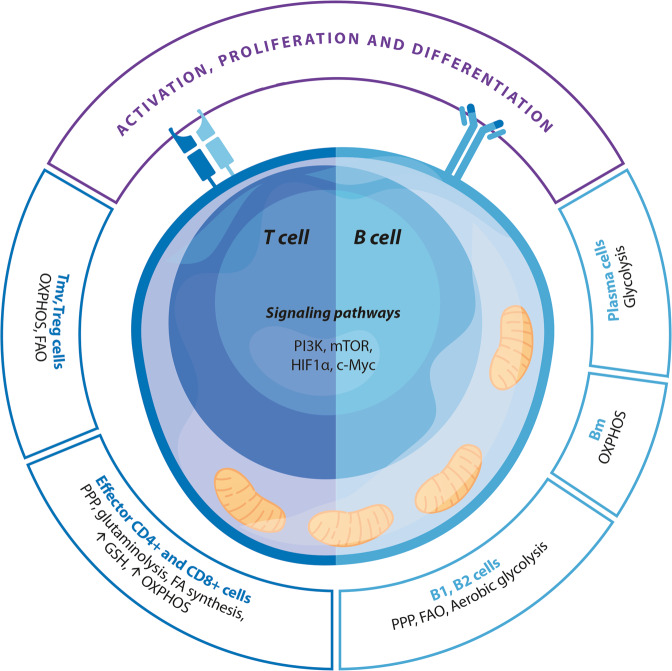
Metabolic reprogramming of T and B cells. Tm cells memory T cells, Treg cells regulatory T cells, OXPHOS oxidative phosphorylation, FA fatty acid, PPP pentose phosphate pathway, GSH glutathione, PI3K phosphatidylinositol 3-kinase, mTOR mechanistic target of rapamycin, HIF1α hypoxia-inducible factor 1-alpha, c-Myc Myc proto-oncogenes, Pl cells plasma cells, Bm cells memory B cells, B1/B2 subclass of B-cells

The metabolic reprogramming of T cells is depicted in Fig. 5 and Table 4 summarizes the redox mechanisms that affect T-cell functions.

**Table 4:** Redox mechanisms influencing T-, B-, and NK-cell functions

Redox mechanisms	T-cell function	References
Reactive oxygen species (ROS)	Encourage Th-2 polarized phenotype	[298]
	Mitochondrial membrane polarization with fatal consequences for T-cell activation and survival following TCR engagement	[300]
	When chronic, may result in T-cell hyperresponsiveness, exhaustion, and anergy	[301–305]
	Dysregulated T-cell homeostasis	[201, 315, 316]
Nuclear factor erythroid 2-related factor 2 (Nrf-2)	Restrains inflammatory T-cell activity	[319, 320]
	Encourages a Th-2 pattern following TCR activation	[317, 318]
Glutathione (GSH)	Suppresses Th-17 differentiation	[321]
	Encourages the production of Tregs	[321]
Thioredoxin (TRX)	Restrains T-cell activation	[326–328]
	Encourages the development of Tregs	
	Enables T effector and Treg cell survival	
Redox mechanisms	B-cell functions	
Mitochondrial reactive oxygen species (mtROS)	Increased ROS inhibit B-cell activation	[353]
	Increased ROS inhibit the differentiation of B-cells into antibody-producing plasmablasts	[351, 352]
	Increased ROS inhibit the production of antibodies by downregulating CD19 expression	[358]
	Increased ROS upregulate the consumption of IgM antibodies	[355–360]
GSH/TRX	Enables medium-term survival	[357]
	Increased production of IgM	[352]
Nuclear factor erythroid 2-related factor 2 (Nrf-2)	Increased survival and increased resistance of ROS-mediated apoptosis	[358–360]
Redox mechanisms	NK-cell functions	
Reactive oxygen species (ROS)	Enable NK-cell-mediated cytolysis	[381]
	Enable NK-cell division and proliferation following pathogen invasion	[382]
Nuclear factor erythroid 2-related factor 2 (Nrf-2)	Restrains activation and regulates effector functions	[383, 384]
Glutathione (GSH)	Enables the proliferation and cytotoxic functions of NK-cells	[385–387]
Thioredoxin 1 (TRX-1)	Maintains membrane cytoprotective sulfhydryl residues in a reduced state	[388, 389]
	Protects cells from hydrogen peroxide-mediated NK-cell dysfunctions	[390–393]

## Metabolic reprogramming and redox regulation of B-cell activation

### Metabolic reprogramming of B-cells

B-cell receptor (BCR) or cytokine-associated activation of naive B cells results in PI3K phospholipase C gamma 1 expression, leading to calcium mobilization and NF-κB activation and upregulation of c-Myc, HIF1α, AKT, mTOR, and STAT-6 [331]. Once activated, these lymphocytes migrate to germinal centers and display high rates of glycolysis and OXPHOS [332–334]. The short-term metabolic reprogramming and increased glycolysis are controlled by PI3K, HIF1α, AKT, and STAT-6 signaling [332–334]. The role of mTOR appears to be confined to the upregulation of GLUT-1 [335]. It is noteworthy that GSK-3 has a key role in regulating glycolysis in activated B cells and may also adjust ROS production and changes in mitochondrial dynamics [335, 336]. However, while mTOR may not be the primary player in the regulation of glycolysis, sustained germinal center B-cell BCR signaling requires activation of mTOR [337, 338]. mTOR is also involved in somatic hypermutation and in the formation of memory B cells [339–341].

The relative levels of OXPHOS and glycolysis differ in plasmablasts and memory B cells, with glycolysis being dominant in the former and OXPHOS being dominant in the latter to enable their long-term survival [342]. B1 and B2 subsets appear to display differing metabolic profiles, with PPP, FAO, and aerobic glycolysis being more active in B1 compared to B2 cells [342]. The production of high-affinity antibodies by plasmablasts is an energetically demanding process and requires rapid increases in glucose consumption and mitochondrial mass accompanied by significant changes in mitochondrial dynamics [336, 343, 344], reviewed in [342]. Unsurprisingly, functional mitochondria are an indispensable element in B-cell differentiation and effector functions [345]. The process of antibody synthesis is also regulated by AMPK, which enables memory B-cell formation and survival in part by regulating mitochondrial dynamics and suppressing the activation of mTOR [133, 346, 347].

### Redox regulation of B-cell activation and function

High levels of hydrogen peroxide are required to initiate and maintain BCR signaling [348, 349]. This is primarily provided by the activity of NOX-2 [350], but in the longer term, the source of hydrogen peroxide is mtROS [348, 349]. In addition, the cellular redox state and mtROS release play a major role in B-cell survival and differentiation and IgM synthesis [351, 352]. However, excessive mitochondrial mtROS synthesis may inhibit B-cell activation and the differentiation of B cells into antibody-producing plasmablasts [353]. Increased  concentrations of mtROS may also inhibit the production of antibodies by downregulating CD19 expression [354]. Finally, chronically upregulated ROS can upregulate the consumption of IgM antibodies [355, 356].

In this context, it is noteworthy that B-cell activation is accompanied by a concomitant stimulation of the TRX and GSH system, with the latter involving triggering of the cystine transporter xCT and higher uptake of cysteine [352]. Upregulation of GSH/TRX systems by activated B cells enables their medium-term survival [357]. The intensive function of both systems correlates with elevated production of IgM [352]. Finally, there is evidence associating increased Nrf-2 expression in activated B cells with prolonged survival and resistance to ROS-mediated apoptosis [358–360].

Table 4 summarizes the redox mechanisms that affect B-cell functions, and the metabolic reprogramming of B cells is depicted in Fig. 5.

## Metabolic reprogramming and redox regulation of NK-cell activation

### Metabolic reprogramming in NK-cells

The signaling mechanisms involved in NK-cell activation [361, 362] entail the engagement of multiple activation receptors such as natural cytotoxicity receptors [363–365] leading to the stimulation of AP-1, NFAT, and NF-κB [361, 366]. Cytoskeletal reorganization and release of chemokines, inflammatory cytokines, and lytic granules containing granzyme A, B, and perforin follows [367–369]. Unsurprisingly, the various effector and regulatory functions of activated NK-cells are enabled by metabolic programming, which is underpinned by the upregulation of glucose-driven glycolysis, OXPHOS, increased FA synthesis, and glutamine metabolism [370–373]. Metabolic reprogramming, glycolysis, and mitochondrial activity are controlled by mTOR that is upregulated in NK cells following stimulation by IL-15 and IL-3 [372, 374, 375]. The high expression of this kinase is also responsible for increased FA synthesis and glutamine metabolism by activated NK cells via the upregulation of SREBPs and N-Myc [370, 376].

In inflammatory conditions, PI3K/mTOR signaling, along with NF-κB and STAT-3 transcriptional activity, is responsible for triggering HIF1 protein synthesis [377, 378]. The importance of mTOR and HIF1α in NK-cell proliferation and function is difficult to overemphasize as reduced HIF1α and mTOR activity are associated with loss of cytotoxic effects. It is evidenced by decreased production of perforin and granzyme B, and premature apoptosis [372, 379, 380].

### Redox regulation of NK-cell activation and function

Increased ROS production enables NK-cell-mediated cytolysis by promoting the release of perforin and granzyme B [381] and NK-cell division and proliferation after pathogen invasion [382]. Nrf-2 activation serves as an immunological checkpoint following NK-cell activation [383, 384].

The upregulation of GSH synthesis may enable the proliferation and cytotoxic functions of NK-cells and, conversely, GSH downregulation results in compromised functions and recruitment to sites of inflammation [385–387]. In an inflammatory environment, the upregulation of TRX-1 plays a role in NK-cell survival by maintaining membrane cytoprotective sulfhydryl residues in a reduced state [388, 389]. This phenomenon may protect those cells from hydrogen peroxide-mediated NK-cell dysfunctions [388, 389]. However, this level of protection is clearly limited as chronic nitro-oxidative stress may result in NK-cell hypofunction and loss of cytotoxic activity [390–393]. There is evidence suggesting that this is due to compromised hydrogen peroxide signaling following NOX-2 hyperactivity [390]. However, there is also proof that NK-cell function may be impaired by excessive production of NO [392].

**Fig. 6:**
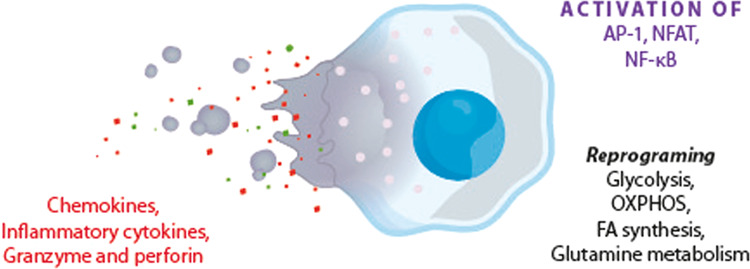
Metabolic reprograming in NK-cells. AP-1 activator protein-1, NFAT nuclear factor of activated T cell, NF-kB nuclear factor NF-kappa-B, OXPHOS oxidative phosphorylation, FA fatty acid

Table 4 summarizes the redox mechanisms that affect NK-cell functions, while Fig. 6 shows the metabolic reprogramming in NK-cells.

## Role of the HDL complex and oxidized phospholipids in the immune response

### Role of HDL, ApoA1, and PON1 in the regulation of the immune response

Previously, we have reviewed the important role of the HDL/ApoA1/PON1 complex in regulating immune responses [13, 41, 79, 90, 394]. In brief, HDL attenuates the activation of TLR-4 by stimulating cholesterol efflux from membrane lipid rafts (MLR), NF-κB activity, DC maturation and activation, and antigen presentation to T lymphocytes. It also affects Th-1 and Th-17 differentiation, T-cell and BCR activation, the complement system, and monocyte and macrophage chemotaxis [13, 41, 79, 90, 394]. HDL-mediated MLR disruption underpins anti-inflammatory and immunosuppressive effects. HDL exerts a unique immunoregulatory role by activating pentraxin 3, an immunosensory molecule. ApoA1 regulates the balance between Th-17 and Tregs, improves mitochondrial functions, increases the activity of the ETC, and stabilizes PON1 within the HDL particle, thereby maintaining PON1 activity. The latter protects against immune cell membrane lipid peroxidation, circulating oxidized lipoproteins, and oxidative damage to mitochondria. It positively affects glucose metabolism, PPP, FAO, PPAR-γ activity, and aerobic glycolysis via upregulation of GLUT-1 [41, 90].

### Role of oxidized phospholipids in the regulation of the immune response

Evidence suggests that the bulk of oxidized phospholipids present in the circulation exists as immune complexes with natural IgM and IgG due to their status as oxidation-specific epitopes or neoantigens [395, 396]. It is also proposed that oxidized phospholipid complexes are proinflammatory [397, 398] using several routes, which include recruitment of the complement cascade [399] and production of inflammatory responses in human macrophages largely by engagement of the Fc gamma receptor 1 [400, 401]. These complexes may activate mature DCs leading to a primed inflammasome thereby exaggerating IFN-γ and IL-1 production [402–404]. Moreover, DCs activated and primed via this mechanism may trigger naive T cells and induce Th-17 polarization [404–406].

As a result of activating neutrophil PRR, oxidized phospholipids contribute significantly to inflammation and oxidative stress and the formation of NETs [407, 408]. In addition, oxidized phospholipid engagement with monocytes, macrophages, DCs, and NK cells may induce epigenetic and metabolic reprogramming leading to “immune training”. The process effectively endows these leucocytes with a *de facto* memory, resulting in an amplified inflammatory or anergic response to future antigenic challenges [409, 410]. The mechanisms driving the metabolic and epigenetic changes described above appear to depend, at least in part, on mTOR-induced assembly of NADPH oxidase and subsequent increases in ROS-mediated signaling [410, 411].

The final part of this review deals with the detrimental effects of chronic oxidative and nitrosative stress on the immune response as a whole. In physiological conditions, NOX-derived cytosolic hydrogen peroxide regulates redox-sensitive intracellular signaling pathways [412–416]. However, in conditions of excessive ROS production, hyperoxidation of thiolate anions to sulfonic acid essentially incapacitates reversible cysteine oxidation. It is an effective signaling mechanism, locking functional cysteines in the oxidized mode [90, 417].

The other signaling system involved in regulating the activity of redox-sensitive proteins and enzymes is reversible S-nitrosylation [17, 418]. However, pathological levels of ROS disable the mechanisms responsible for maintaining the reversibility of S-nitrosylation inducing a cellular state described as protein hypernitrosylation [202]. Hyperoxidation and S-nitrosylation can result in impaired function of the redox-sensitive transcription factors and enzymes regulating metabolic reprogramming in immune cells. Compromised mitochondrial functions and seriously suppressed immune cell activation and function may follow. Chronic nitro-oxidative stress also affects the activity of HDL, apoA1, and PON1 whilst increasing the density of oxidized phospholipids further dysregulates the immune response [41]. Finally, chronic nitro-oxidative stress and inflammation also stimulate IDO that may result in a state of profound immune suppression [419]. The section below deals with these processes, beginning with the effects of hypernitrosylation and hyperoxidation on transcription factors and enzymes.

## The detrimental effects of chronic nitro-oxidative stress on the immune response

### Chronic nitro-oxidative stress on transcription factors and enzymes

S-nitrosylation exerts a significant inhibition of NF-κB function by reducing the binding of its subunits to DNA thereby decreasing the activity of the complex as a transcription factor [420–422], as well as the expression of target effector genes [420, 423]. This consequence is largely due to S-nitrosylation-mediated conformational changes to crucial functional cysteine residues located on the p65 subunit of p50/p65 abrogating NF-κB DNA-binding capacity [420, 424]. The outcomes involve decreased levels of IL-12 [425], IL-1β [426], IL-6, IL-8, and iNOS [427, 428]. Moreover, S-nitrosylation may inhibit TLR-4 [429, 430] and TLR-2 signaling [431].

There is also in vivo evidence that S-nitrosylation leads to the inhibition of numerous MAPKs, most notably p38/MAPK [432, 433], Janus kinase [432, 434], and consequent STAT-3 and NF-κB activation [435]. S-nitrosylation is additionally involved in Nrf-2 triggering, which appears to be affected via the conformational modification of crucial thiol groups [436–438]. Hypernitrosylation is also accompanied by chronic activation of HIF1α via upregulation and/or stabilization of HIF1α [439–441]. In addition, irreversible nitrosylation of functional cysteine thiols may cause chronic upregulation of PI3K/AKT and mTOR signaling [442–445] thereby decreasing the capacity of immune cells to adapt to environmental conditions or changing metabolic needs. Moreover, mTOR may be directly activated following *S*-nitrosylation of the tuberous sclerosis complex 2 [445] and the nitrosylation of small GTPases [446]. Prolonged nitrosylation may also compromise immune cells via the chronic upregulation of GSK-3 [202]. Finally, by inhibiting AMPK activity, nitrosylation-mediated upregulation of PI3K/AKT and GSK-3 may introduce a further dimension of metabolic disorders [447, 448]. In addition, in an environment of chronic nitro-oxidative stress, mTOR may be inactivated by oxidation of Cys1483 [449] and AMPK activation [450, 451]. In an environment of increased ROS, several enzymes involved in regulating metabolic reprogramming in immune cells are triggered most notably via PPAR-γ [452, 453].

### Detrimental effects on immune cells due to nitro-oxidative stress-mediated mitochondrial dysfunction

Chronically elevated ROS/RNS can impair mitochondrial structure and functions by injuring DNA, proteins, and lipids. The most prominent results are damage to the enzymes of the ETC [248, 454–456] and a range of structural and functional phospholipids, basically cardiolipin [457–459]. This ultimately leads to altered ATP production and accelerated ROS, provoking further impairement of macromolecules, forming the basis of self-amplifying pathology [248, 454–456]. Increased NO production by mitochondria in an environment of nitrosative stress may also be a source of dysfunction and damage [460–462]. In essence, two pathways are implicated. The first involves reversible inhibition of ETC enzymes by NO-mediated S-nitrosylation [17, 463, 464]. The second comprises irreversible nitration of functional enzymes and structural proteins by ONOO^-^ [248, 465]. This pattern of pathology leads to a vicious circle of bioenergetic failure and elevated mtROS production [466–469].

Clearly compromised mitochondrial function has many direct adverse effects on the activity of immune cells, as discussed above. However, mitochondrial dysfunction may also lead to numerous indirect negative consequences related to depleted levels of NADPH, which results from the distorted activity of this organelle [470–472]. This is a significant source of metabolic dysfunction in immune cells as the GSH/TRX systems are wholly dependent on the presence of adequate levels of NADPH, which acts as an indispensable source of reducing equivalents [473–476]. The synthesis of NADPH from NADP [477, 478] and NAD^+^ kinases, which catalyze the production of NADP from NAD^+^ [479, 480], is dependent on mitochondrial respiration and on an adequate supply of ATP [470, 471, 481]. Mitochondrial dysfunction is associated with depleted levels of NAD^+^ [13] due to the fact that the enzyme nicotinamide mononucleotide adenylyl transferase, which catalyzes the formation of NAD^+^ synthesis from nicotinamide mononucleotide as part of the salvage pathway [482], is dependent on ample supplies of ATP [483–485].

An important adverse consequence of depleted NAD^+^ levels is the compromised mitochondrial NADPH production by malic enzyme 2, IDH, methylenetetrahydrofolate dehydrogenase 2, and aldehyde dehydrogenase, which are all NAD^+^ dependent [486, 487]. Lowered levels of malic enzyme 2 and IDH may affect the TCA cycle [488, 489]. NAD^+^ deficiency can impair the PPP's ability to produce NADPH via decreased hexokinase activity [490–492].

### Chronic nitro-oxidative stress and the inhibition of antioxidant systems and TCA activity

Chronic nitro-oxidative stress may cause nitrosylation and hyperoxidation of the key cysteine residues within TRX and thioredoxin reductase thereby compromising or abrogating TRX activity [493–496]. Chronically elevated ROS/RNS decrease GSH system activity [497, 498]. Mechanistically, this is achieved via the oxidation and nitrosylation or tyrosine nitration or via inhibiting the activity of GSH, glutathione peroxidase, and glutathione reductase [13, 321, 499]. Increased production of radical species also raises the activity of multidrug resistance-associated proteins, resulting in extrusion of GSH and GSSH into the intercellular environment. The decreased importation of cysteine, which follows, leads to reduced synthesis of replacement GSH [500–503]. A state of persistent nitro-oxidative stress may also cause Nrf-2 inhibition via several mechanisms, including activation of MAPK kinase, decreased DJ-1 [459, 504], and reduced TRX system activity [505, 506].

Oxidation and/or nitrosylation of functional cysteine groups in several TCA enzymes may cause adverse effects on the metabolism of immune cells. Such inactivated enzymes are α-ketoglutarate dehydrogenase [507–509] and conitase, which catalyze the conversion of citrate to isocitrate [510, 511], IDH [512–514], ME2 [515, 516], and pyruvate dehydrogenase kinase [517]. The negative consequences of lowered α-ketoglutarate dehydrogenase and aconitase are of particular importance, and may lead to reduced TCA cycle activity and NADPH synthesis [518, 519] and accumulation of citrate [519]. The inactivation of pyruvate dehydrogenase kinase also results in adverse metabolic consequences by attenuating the conversion of pyruvate to acetyl-CoA [517].

### Detrimental effects of chronic nitro-oxidative stress on the HDL complex

Chronically elevated ROS/RNS levels are a cause of depleted circulating HDL [520–522], ApoA1 [522–524], and PON1 [525, 526] levels. Chronic oxidative stress induces HDL [527–529] and ApoA1 [521, 530, 531] dysfunctions. PON1 is rendered dysfunctional in such an environment, which appears to be mediated by the high activity of MPO [525, 526, 532]. The mechanisms underpinning the development of a dysfunctional HDL particle and reduced activity of ApoA1 are complex and readers are referred to the work of Morris et al. [41].

### Chronic nitro-oxidative stress and the advent of immunosuppression

Chronic nitro-oxidative stress can induce the development of endotoxin tolerance by provoking IDO activation [533, 534]. Increased IDO activity upregulates the tryptophan catabolite (TRYCAT) pathway, as well as TGF-β1 and IL-10 [535, 536], which exert multiple inhibitory effects on TLR signaling [537, 538]. Neutrophils with endotoxin tolerance are characterized by decreased oxidative burst, downregulated TLR-4 receptors, and impaired cell adhesion, rolling, and migration [539–541]. Macrophages with endotoxin tolerance display significant dysregulation of their function as APCs [542]. Impaired antigen presentation is also seen in DCs following IDO activation [542]. In this state, DC activation of naive T cells leads to Th-2 polarization [543, 544]. DCs may inhibit T memory and T effector cells and induce CD4 and CD8 T-cell anergy and activation of Tregs [545, 546]. This explains that prolonged endotoxin tolerance is typified by impaired proliferation and anergy of CD4 T and CD8 T cells and increased Treg cell numbers [547–549]. Finally, endotoxin tolerance is characterized by a reduced number and cytolytic function of NK cells [550–552].

## Summary and conclusion

The functions, performance, and survival of immune cells are strongly regulated by redox mechanisms, including intracellular and extracellular ROS/RNS and oxidized phospholipids, cellular antioxidants such as glutathione, thioredoxin, the HDL complex, and Nrf-2. Hypernitrosylation and chronic nitro-oxidative stress may inhibit these antioxidant systems, thereby decreasing the activity levels of the TCA cycle, mitochondrial functions, and immune cell metabolism. As such, redox mechanisms regulate and modulate many different immune functions, including but not limited to macrophage and Th cell polarization, phagocytosis, production of pro- and anti-inflammatory cytokines, metabolic reprogramming of immune cells, immune training and tolerance, chemotaxis, pathogen sensing, antiviral and antibacterial effects, TLR activity, and endotoxin tolerance. ROS/RNS, oxidized phospholipids, and the key antioxidant systems could be regarded as new drug targets in the treatment and prevention of immune disorders.
